# Multi-focal evaluation and establishment of primary care for recently incarcerated women

**DOI:** 10.1186/1940-0640-10-S1-A41

**Published:** 2015-02-20

**Authors:** Diane S Morse, John L Wilson, Ann M Dozier, Catherine Cerulli

**Affiliations:** 1 Department of Psychiatry, University of Rochester School of Medicine, Rochester, NY, 14642, USA; 2 Department of Medicine, University of Rochester School of Medicine, Rochester, NY, 14642, USA; 3 Department of Public Health Sciences, University of Rochester School of Medicine, Rochester, NY, 14642, USA; 4 Susan B. Anthony Center for Women’s Leadership, University of Rochester, Rochester, NY, 14627, USA

## Background

Individuals recently released from incarceration face challenges while accessing primary medical care, mental health care, addiction treatment, and medication [1]. Furthermore, women are the fastest growing incarcerated population and have additional health risks, such as histories of trauma, high-risk sexual behaviors, and increased risk of contracting HIV and hepatitis C [2]. Re-entering individuals often resort to emergency rooms, where they will not obtain adequate long-term strategies for treatment. Primary care clinics have been established for patients with substance abuse [3], with recent recommendations to add mental health care [2]. We examined effects of a transitions primary care clinic for recently released women housed in a department of psychiatry at an academic medical center.

## Materials and methods

Formerly incarcerated community health workers (CHWs) recruited women (N = 95) recently released and scheduled for release from jail, prison, probation, or transitional housing. Women who lacked an adequate primary care provider were recruited to attend the Women’s Initiative Supporting Health (W.I.S.H.) Transitions Clinic between September 2012 and July 2014. W.I.S.H. is one of 11 culturally informed clinics within the Transitions Clinic Network consortium and employs trauma and culturally informed practices [4]. An internist (DSM) conducted comprehensive, multifocal evaluations on all clinic patients. The key aim of this project was to assess the extent to which screening and assessment resulted in patient follow-up recommendations for future testing. Of primary interest were testing for HIV, hepatitis A, B, and C, and sexually transmitted infections.

## Results

Of the 95 women recruited (Table 1), 68 (72%) attended the clinic at least once and completed the intake process. Women were recruited at the local jail (n = 26), transitional housing (n = 21), community supervision programs (n = 12), shelters (n = 5), community agencies (n = 2), and through self-referrals (n = 2). The majority of patients who were referred to testing completed the testing (Table 2). Patients received mental health and addiction assessments (including nicotine) and were offered treatment.

**Table 1:** Demographical information of individuals recruited from September 2012 to July 2014

	Attended clinic		Did not attend clinic	
	
	N	Percentage	N	Percentage
Number of individuals recruited	68	-	27	-
Mean age (SD)	37.3 (11.1)	- -	34.4 (10.2)	- -
Race: African American Caucasian Asian Other	26 36 1 5	38 53 2 7	14 13 0 0	52 48 0 0
Ethnicity: Hispanic	6	9	22	81
History of intimate partner violence	42	62	-	-
History of child abuse	19	28	-	-

**Table 2:** Women’s Initiative Supporting Health (W.I.S.H.) transitions clinic patient testing data from September 2012 to July 2014

	N	Percentage
Hepatitis A testing Testing indicated/ordered Completed testing	62 39	91 72
Hepatitis B testing Testing indicated/ordered Completed testing	67 45	98 75
Hepatitis C testing Testing indicated/ordered Completed testing	63 42	92 71
HIV testing Testing indicated/ordered Completed testing Declined testing	66 43 2	97 70 3
Sexually transmitted infection testing Testing indicated/ordered Completed testing	40 36	59 90

## Conclusions

Women recently released from incarceration to a clinic housed in psychiatry succeeded in linking patients to primary care and assessments. Formerly incarcerated CHWs recruited most women from incarceration and transitional housing. Clinics for justice-involved women using a trauma-informed approach may serve to improve these vulnerable patients' health, which in turn may improve the health of their families and communities. More study is needed to address potential policy changes required in hiring those with a felony history. Additionally, efforts to engage Hispanic women and those who declined testing are worthy of further explanation.
